# Identification of FN1BP1 as a Novel Cell Cycle Regulator through Modulating G1 Checkpoint in Human Hepatocarcinoma Hep3B Cells

**DOI:** 10.1371/journal.pone.0057574

**Published:** 2013-02-28

**Authors:** Mei Liu, Ronghua Wu, Fuye Yang, Tao Wang, Pingping Zhang, Jianren Gu, Dafang Wan, Shengli Yang

**Affiliations:** 1 The Jingsu Key Laboratory of Neuroregeneration, Nantong University, Nantong, Jiangsu, China; 2 State Key Laboratory of Oncogenes and Related Genes, Shanghai Jiaotong University, Shanghai, China; Virginia Commonwealth University, United States of America

## Abstract

A novel human gene, *FN1BP1* (fibronectin 1 binding protein 1), was identified using the human placenta cDNA library. Northern blotting showed a transcript of ∼2.8 kb in human placenta, liver, and skeletal muscle tissues. This mRNA transcript length was similar to the full *FN1BP1* sequence obtained previously. We established a conditionally induced stable cell line of Hep3B-Tet-on-FN1BP1 to investigate the preliminary function and mechanism of the secretory FN1BP1 protein. Cell-proliferation and colony-conformation assays demonstrated that FN1BP1 protein suppressed Hep3B cell growth and colonization *in vitro*. Analysis of Atlas human cDNA expression indicated that after *FN1BP1* Dox-inducing expression for 24 h, 19 genes were up-regulated and 22 genes were down-regulated more than two-fold. Most of these gene changes were related to cell-cycle-arrest proteins (p21cip1, p15, and cyclin E1), transcription factors (general transcription factors, zinc finger proteins, transcriptional enhancer factors), SWI/SNF (SWItch/Sucrose NonFermentable) complex units, early-response proteins, and nerve growth or neurotrophic factors. Down-regulated genes were subject to colony-stimulating factors (e.g., GMSFs), and many repair genes were involved in DNA damage (RAD, ERCC, DNA topoisomerase, polymerase, and ligase). Some interesting genes (*p21cip1*, *ID2*, *GMSF*, *ERCC5*, and *RPA1),* which changed in the cDNA microarray analysis, were confirmed by semi-qRT-PCR, and similar changes in expression were observed. FCM cell-cycle analysis indicated that *FN1BP1* over-expression could result in G1 phase arrest. FN1BP1 might inhibit cell growth and/or colony conformation through G1 phase arrest of the Hep3B cell cycle. These results indicate the potential role of FN1BP1 as a treatment target for hepatocellular carcinoma.

## Introduction

Hepatocellular carcinoma (HCC) ranks among the most common cancers in the world and is one of the leading causes of cancer-related death. In the past three decades, the age-adjusted incidence of liver cancer has risen from 16 per million individuals to 46 per million individuals, with the greatest increases occurring in Asia and Africa [1]. The carcinogenesis and progression of HCC has an etiology that includes multiple steps and multiple gene involvement. To date, many genes involved in HCC occurrence, development, and progression have been comprehensively studied in depth, including *p53*, *p21* [2], *p16*, beta-catenin, *PTEN*, and *Rb*. Recent studies of HCC using functional gene screening have further revealed that a number of genes with novel sequences and unclarified functions are related to HCC development or progression [3]. In 2004, a direct genome-wide functional screening method based on large-scale cDNA transfection was established by Wan et al. [4]. This screening method facilitates the compilation of a broad and comprehensive database of genes that may affect cancer formation and progression. Since then, many functional genes related to cell growth and cancer development and progression have been identified and further investigated, such as *BNIPL-2* [5], *CPGL-B* [6], *Aph2* [7], *CT120A* [8], *POL λ2* [9], to name a few. Among these, a human novel gene, *FN1BP1* (also named pp1195 in GenBank, Accession No. AF217970), which encodes a protein composed of 147 amino acids with a predicable signal peptide, was first identified using this method at the National State Key Laboratory for Oncogenes and Related Genes. Bioinformatic analysis showed that the *FN1BP1* gene is located on chromosome 8p23.1. In a preliminary two-yeast hybridization study, we found that the gene interacted with fibronectin (FN) and promoted the cell migration [10]. In the present study, we established a Hep3B cell line with the doxycycline-regulated FN1BP1 transgenic system to provide additional evidence for the function and preliminary mechanism of FN1BP1 protein.

## Materials and Methods

### 1 Northern Blot Hybridization for *FN1BP1* Gene Expression on Multiple Tissues

Agarose gel–purified PCR products (primers shown in Table 1) were used as northern blot analysis cDNA probes and were labeled with α-^32^P-dCTP (10 µCi/µl, Amersham Life Sciences, Arlington Heights, IL, USA) using a Random Primed DNA Labeling Kit (Roche, Basel, Switzerland). A Quick Spin Column of Sephadex G-50 was used to remove the unincorporated deoxyribonucleoside triphosphates. The denatured labeled cDNAs were probed to human MTN Blot (Multiple Tissue Northern Blot, 8-lane, Clontech, Mountain View, CA, USA) in 5 ml ExpressHyb™ hybridization solution (Clontech) supplied with sheared, denatured DNA from salmon sperm (Sigma-Aldrich, St. Louis, MO, USA) according to the manufacturer’s instructions. The blots were washed, and auto-radiograms were developed after exposure to X-ray film (Kodak, X-Omat) at −70°C.

**Table 1:** Primers used in this study.

Target gene	Sequence (5′- to -3′)	Position	Product
FN1BP1-sense	tgcttgtctgatgactgatgg	nt2070∼nt 2092	480 bp, probe for northern blot
FN1BP1-antisense	agaagggaggagttgctcaa	nt 2551∼nt 2532	
FN1BP1 (tet)-up:	gca ggatcc ggcatgggcttgctttctc	nt 336∼nt 353	FN1BP1 sense, 500 bp for pTRE2hyg. HA tag is italic.
FN1BP1 (tet)-down	agc atcgat *agcgtaatctggaacgtc* *atatggata*ttgggacacaacacactc	nt 779∼nt 763	
FN1BP1 (tet)-anti-up	gca atcgat ttaggcatgggcttgctttc	nt 336∼nt 353	FN1BP1 antisense, 500 bp for pTRE2hyg. HA tag is italic.
FN1BP1 (tet)-anti-down	agc ggatcc *agcgtaatctggaacgtc* *atatggata*ttgggacacaacacactc	nt 779∼nt 763	
FN1BP1-HA-sense	ccgctcgagggcttgctttctcta	nt 336∼nt 353	443 bp for pcDNA-HA-FN1BP1
FN1BP1-HA-antisense	gctctagattgggacacaacaca	nt 779∼nt 763	
FN1BP1 (3.1)-sense	ggcatgggcttgctttctct	nt 331∼nt 349	448 bp for pcDNA3.1/His-V5-FN1BP1
FN1BP1 (3.1)-antiense	ttgggacacaacacactcat	nt 779∼nt 763	
1195(GFP)-sense	ggaattcgaggcatgggcttgctttc	nt 336∼nt 353	456 bp for pEGFP-N1-FN1BP1
1195(GFP)-antiense	cgggatcctgggacacaacacactcatt	nt 779∼nt 763	
p21-sense	gtcaccgagacaccactgga	nt 254∼nt 273	300 bp for p21^cip1^
p21-antisense	cggcgtttggagtggtaga	nt 561∼nt 543	
ID2-sense	aaaacagcctgtcggaccac	nt 143∼nt 162	323 bp for ID2
ID2-antisense	ttcagaagcctgcaaggaca	nt 465∼nt 446	
GMSF-sense	ggcgtctcctgaacctgagt	nt 151∼nt 170	296 bp for GMSF
GMSF-antisense	gcagtcaaaggggatgacaa	nt 446∼nt 427	
ERCC5-sense	gcagccagcgaaatagaagc	nt 3136∼nt 3155	302 bp for ERCC5
ERCC5-antisense	tgcgaatctgaagcactggt	nt 3438∼nt 3418	
RPA1-sense	gctgtgagaagtgcgacacc	nt 1565∼nt 1584	285 bp for RPA1
RPA1-antisense	cgtccatcacagtggcctta	nt 1849∼nt 1883	
β-actin -sense	atcgtgatggactccggtga	nt 523∼nt 542	493 bp for β-actin
β-actin -antisense	ttctgcatcctgtcggcaat	nt 1016∼nt 977	

### 2 Plasmid Construction, Cell Culture, and Stable Transfection

Procedures were identical to those followed in our previous study [11]. Briefly, for the construction of plasmid pTRE2hyg-FN1BP1, the ORF (open reading frame) sequence of *FN1BP1* was amplified by PCR using the primers containing BamH I and Cla I restriction sites and an HA (hemagglutinin)-tag sequence (Table 1). These sequences were cloned into the linearized Tet-On expression vector of pTRE2hygc (Clontech), which contains the hygromycin resistance gene. The recombinant plasmid, pTRE2hyg-FN1BP1, was confirmed by DNA sequencing. Cell culture, plasmid transfection, and western blot analysis were conducted following the methods reported in previous studies [5], [6], [7], [8], [9]. The Tet-On Hep3B cells, established by Dr. Wang [11], [12] and stored in our lab, were maintained in Dulbecco’s modified Eagle’s medium (DMEM, Gibco, Invitrogen, Grand Island, NY, USA) supplemented with 15% Tet-system–approved fetal bovine serum (Clontech), 50 mg/ml G418, penicillin (100 U/ml), and streptomycin (100 µg/ml) at 37°C in a humidified 5% CO_2_ incubator. The pTRE2hyg-FN1BP1 DNA was transfected into the Tet-On Hep3B cells using LipofectAMINE (Invitrogen). The stable cell populations were selected by incubation in the media containing hygromycin (0.1 mg/ml) (Invitrogen) and were allowed to form colonies and further expand. After selection in the medium containing 25 mg/L hygromycin for more than 8 wk, these colonies were analyzed by western blotting. The cells of each clone were induced by Dox (2 µg/ml, a tetracycline analogue) for 24 h to express FN1BP1, followed by lysis in T-PER® Tissue Protein Extraction Reagent (Pierce, Thermo Scientific, Rockford, IL, USA) with protease inhibitor on ice. Total proteins of the whole-cell lysates quantified with a BCA kit (Pierce) were resolved by 15% SDS-PAGE and transferred to a nitrocellulose transfer membrane (PROTRAN®, Schleicher & Schuell Bioscience, Keene, NH, USA). After they were blotted with the anti-HA antibody (Sigma) or anti-FN1BP1 antibody (CASB Biotechnology, Shanghai, China) and the corresponding secondary antibodies (Sigma), the immunoblots were developed using enhanced chemiluminescence reagents SuperSignal® West Femto Maximum Sensitivity Substrate (Pierce) and X-OMAT imaging film (Kodak).

### 3 Subcellular Localization of *FN1BP1* Protein

The ORF sequence of *FN1BP1* was transfected into the pEGFP-N1 (Clontech) vector and the pEGFP–FN1BP1 construct was identified by DNA sequencing. The Hep3B cells in a 24-well Nunc plate were transfected with 1 µg of pEGFP or pEGFP–FN1BP1, respectively, using 2 µl of lipofectAMINE2000 (Invitrogen) per well according to the manufacturer’s instructions. After 24 h, the transfected cells were digested using 0.25% trypsin and left to expand in a 35-mm dish. After an additional 24 h of growth, the transfected cells were photographed using an inversion fluorescence microscope (Leica Microsystems, Wetzlar, Germany).

### 4 Investigation the Secretion Characteristic of the FN1BP1 Protein

To investigate whether the *FN1BP1* gene is a secreted protein, we constructed the recombinant plasmid pcDNA3.1-FN1BP1 to transfect into Hep3B cells, in parallel with pcDNA3.1 transfected cells as control. Three days later, the supernatant of culture media from transfected cells in each group was collected to investigate whether the FN1BP1 was secreted into culture media by western blotting using anti-FN1BP1 antibody. The procedures of transfection and western blotting were described in the previous section.

### 5 Determination of Cell Proliferation Activity in vitro

To measure cell proliferation, the Hep3B Tet-On FN1BP1/S11, which was identified as an induced positive colony expressing FN1BP1, was expansively cultured, and then cells were trypsinized and plated to 96-well plates at equal densities. The following day, half of the cells were cultured in an inducible medium containing Dox (2 mg/mL). Media were replaced once every 2 days to maintain inducible conditions. A Cell Counting Kit-8 (CCK-8, Dojindo Laboratories, Kumamoto, Japan) was used to examine cell proliferation activity once daily for 6 days according to the manufacturer’s protocol and previous studies [13]. Measurements were repeated at least three times.

### 6 Cell Colony Formation Assay

The FN1BP1/S11 cells were plated in a six-well plate with 1 × 10^3^ cells per well. The following day, half of the wells were changed with Dox^+^-inducing media to maintain the inducible condition. After 2 weeks, the media were removed and the cells were fixed gently for 15 min with 10% acetic acid/10% methanol. Then the cells were stained with 0.4% crystal violet in 20% ethanol for 15 min to visualize the colonies [6], [8]. The same assays were performed at least three times.

### 7 Human cDNA Expression Arrays and Semi-quantitative RT-PCR

The experiments were conducted according to the manufacturer’s instructions and previous literature [13], [14]. The FN1BP1/S11 cells were cultured and expanded into four 10-cm dishes to 70% confluence, and two were treated with Dox for 24 h. Then total RNA samples were extracted using TRIzol reagent (Gibco). Before labeling, total RNA of each sample was treated with DNase I (TaKaRa) to remove contaminated DNA. Five µg of total RNA was used to perform the labeling reaction with [α-^32^P] dATP (10 µCi/µl, Amersham Life Sciences) strictly according to the manufacturer’s instructions (Clontech). The first strands of cDNA probes were labeled, purified, denatured, and then used in hybridization. Membrane hybridization (Atlas human cDNA expression arrays; Cat No. 7740-1, Clontech) and exposure were performed as mentioned in the previous section. The images were scanned using a Cyclone™ storage phosphor system (Packard Bioscience, Meriden, CT, USA) and analyzed using a Quantarray® image system (Packard Bioscience). Housekeeping genes ubiquitin and b-actin were selected for normalization. The normalized intensity of each spot representing a unique gene expression level was acquired. Genes were considered to be up-regulated when the intensity ratio was >2 and down-regulated when the intensity ratio was <0.5 [8], [11]. To check the cDNA array results, five genes, *p21^cip1^* (cyclin-dependent kinase inhibitor), *ID2* (inhibitor of DNA binding 2), *GMSF* (granulocyte-macrophage colony stimulating factor), *ERCC5* (excision repair cross-complementing rodent repair deficiency, complementation group 5), and *RPA1* (replication protein A1) were selected for confirmation by semi-quantitative RT-PCR with β-actin as an internal control. Briefly, 5 µg of total RNA extracted by TIRZOL Reagent (Invitrogen) from induced or non-induced cells were reverse-transcribed into 20 µl of the first strand cDNA using the SuperScript™ (Invitrogen) first-strand synthesis system, and then 1 µl of each product was used as the template to amplify each specific gene fragment in 25 µl reaction mixture with corresponding primers (Table 1). Ten µL of PCR reaction products were analyzed by electrophoresis of agarose gel and visualized by ethidium bromide staining.

### 8 Cell Cycle Determination by Flow Cytometry

The FN1BP1/S11 cells were trypsinized and planted in two 35-mm culture dishes at equal densities. The following day, cells were synchronized using nocodazole (Sigma) [8], [14]. After the nocodazole was withdrawn, Dox was added to one of the plates to induce the expression of FN1BP1 for 24 h. Then the cells were irradiated under UV at 200 (×100 µJ/cm^2^) for 1 min [8], [15], [16]. The UV-treated cells were collected after an additional 12 h of incubation. Then the cells were washed with PBS, fixed with 70% ethanol, and cooled to −20°C overnight. For flow cytometry (FCM) analysis following the method described in previous studies [6], [14], the ethanol-fixed cells were digested by 1% RNase A (ribonuclease A, Sigma) for 30 min, stained with propidium iodide (PI) at 4°C, protected from light, filtered twice, then measured with a flow cytometer (FACS Calibur, Becton Dickinson, Franklin Lakes, NJ, USA).

### 9 Statistics

All data analyses, statistical comparisons, and graphs were generated using Microsoft® Excel® (Microsoft, Redmond, WA, USA). Data represent mean ± SD of three or four separate experiments for each assay, and comparisons were performed using a two-tailed *t* test or univariate ANOVA. For all statistical analyses, the mean difference was considered to be significant at the p<0.05 level.

## Results

### 1 *FN1BP1* Expression in Multiple Tissues

**Figure 1:**
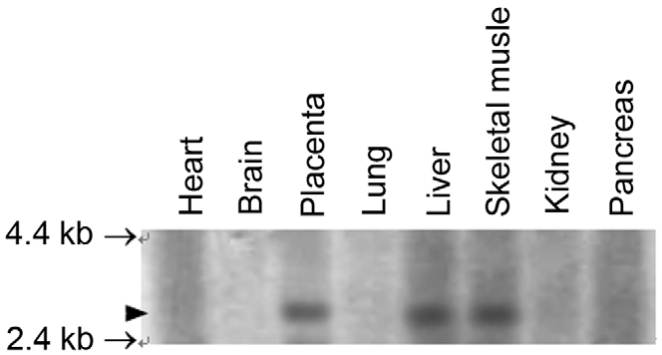
FN1BP1 expression in human placenta, liver, and skeletal muscle tissues. The tissue expression pattern of FN1BP1 (Accession No. AF217970) was investigated by hybridizing its cDNA probe to a multiple-tissue northern blot assay. The mRNA content in the eight lanes are from heart, brain, placenta, lung, liver, skeletal muscle, kidney, and pancreas tissues, respectively. A transcript of ∼2.8 kb was observed in human placenta, liver, and skeletal muscle tissues. The arrow (no tail) shows this ∼2.8-kb length of the mRNA transcript. The arrows at 2.4 kb and 4.4 kb indicate the mean sizes of the RNA markers.

The tissue expression pattern of FN1BP1 was investigated using multiple-tissue northern blotting. Figure 1 shows the result of northern blotting for human MTN using the FN1BP1 probe. A ∼2.8-kb fragment was observed in human placenta, liver and skeletal muscle tissues. This mRNA transcript length was similar to the full FN1BP1 sequence we obtained previously.

### 2 FN1BP1 is a Secreted Protein with a Signal Peptide Localized in the Cytoplasm of Hep3B Cells in vitro

**Figure 2:**
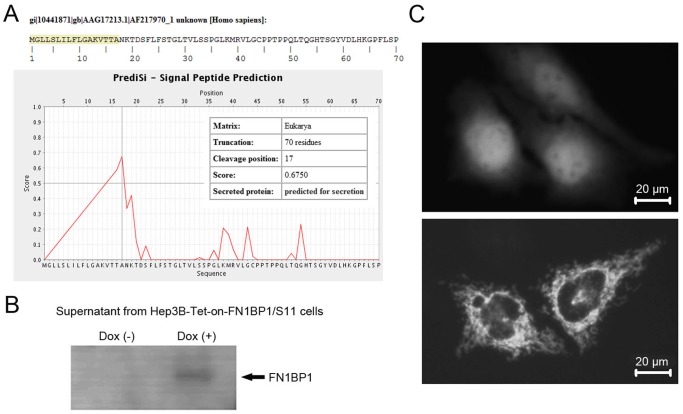
FN1BP1 is a secreted protein localized in the cytoplasm of Hep3B cells in vitro. (A) The FN1BP1 ORF was postulated to be a secreted protein with a signal peptide (SignalP software). To investigate whether the *FN1BP1* gene is a secretory protein, we collected the supernatant of culture media from pcDNA3.1-FN1BP1 transfected cells to perform western blotting using anti-FN1BP1 antibody. (B) Compared with the supernatant of the media-cultured pcDNA3.1-transfected cells, FN1BP1 protein could be detected in the supernatant from the media of pcDNA3.1-FN1BP1-transfected cells by western blot analysis. To investigate the localization of FN1BP1 protein, the pEGFP-FN1BP1 recombinant plasmid was constructed and transfected into Hep3B cells. (C) After transfection for 48 h, the Hep3B cells were observed and the FN1BP1 protein-fused GFP accumulated in the cytoplasm. In the upper panel of Fig. 2C, control GFP is ubiquitously expressed in both nucleus and cytosol, while in the lower panel of Fig. 2C, GFP-FN1BP1 is expressed only in cytosol.

The full-length ORF sequence of FN1BP1 was postulated to be a secreted protein with a signal peptide using SignalP software (Fig. 2A). To investigate whether the FN1BP1 gene is a secretory protein, we collected the supernatant of culture media from pcDNA3.1-FN1BP1-transfected cells to perform western blotting using anti-FN1BP1 antibody. Compared with the supernatant of the media-cultured pcDNA3.1 transfected cells, FN1BP1 protein was detected in the supernatant from the media of pcDNA3.1–FN1BP1-transfected cells by western blot analysis (Fig. 2B). To investigate the localization of FN1BP1 protein, the pEGFP–FN1BP1 recombinant plasmid was constructed and transfected into Hep3B cells. Forty-eight hours after transfection, the Hep3B cells were observed (Fig. 2C). The FN1BP1 protein-fused green fluorescent protein (GFP) accumulated in the cytoplasm.

### 3 FN1BP1 Reduced Cell Proliferation and Suppressed Cell-colony Formation on the Induced Stable Hep3B Tet-On FN1BP1/S11 Cells

**Figure 3:**
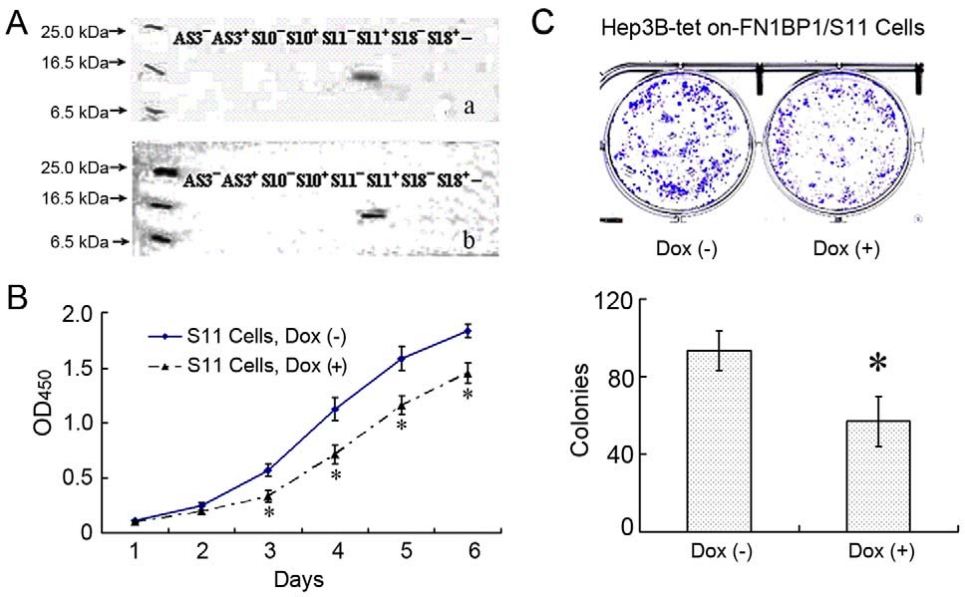
Inducing FN1BP1 expression in Hep3B cells reduced cell proliferation and colony formation. After screening in the medium containing hygromycin for more than 8 wk, a total of 33 independent Hyg-resistant cell lines were obtained from the Hep3B-Tet-on cells transfected with pTRE2hyg-FN1BP1. (A) The 11th sense clone of Hep3B Tet-On-FN1BP1 (marked as Hep3B-Tet-On-FN1BP1/S11) showed a positive band identified by either anti-HA tag antibody (a) or anti-FN1BP1 antibody (b) when the cells were induced by Dox. S10 and S18 represent the 10th and 18th sense clones of Hep3B Tet-On-FN1BP1. AS3 represents the third antisense clone of Hep3B Tet-On-FN1BP1, respectively. (B) The cell proliferation activity curve of 6 days shows that expression of FN1BP1 suppressed the Hep3B cell proliferation activity significantly from day 3 to day 6. The data are represented as mean ± SD, * P<0.05 vs non-induced S11 cells, n = 4. (C) The data indicate that the expression of FN1BP1 induced by Dox distinctly decreased the cell colony formation compared to non-induced Hep3B cells, *P<0.05, n = 4. The upper panel shows the representative figure, while the statistical result is shown in the lower panel.

After it was screened in the medium containing hygromycin for more than 8 wk and identified by western blotting, the 11th clone (named the Hep3B Tet-On FN1BP1/S11 cells) showed a positive band while the cells were induced by Dox using either HA tag antibody or FN1BP1 polyclonal antibody (Fig. 3A). The cell proliferation activity of Hep3B Tet-On FN1BP1/S11 cells with or without Dox treatment is shown in Fig. 3B, the data demonstrate that the over-expression of FN1BP1 suppressed Hep3B cell growth. A marked decline in growth began on the third day of culture. In the colony formation assay, the number of colonies in the group of Hep3B Tet-On FN1BP1/S11 cells with Dox induction was greatly decreased (Fig. 3C) compared to the non–Dox-induced Hep3B cells. These results prove that FN1BP1 can repress cell colony formation.

### 4 FN1BP1 Resulted in Gene Expression Profiles that Show Alteration of Hep3B Cells

**Figure 4:**
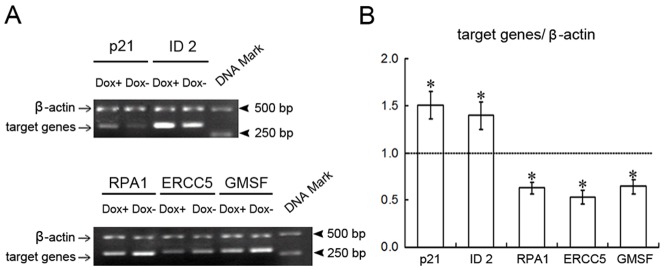
The semi-quantitative RT-PCR analysis of p21^cip1^, ID2, GMSF, ERCC5 and RPA1. (A) The mRNA levels of five genes, *p21^cip1^, ID2, GMSF, ERCC5*, and *RPA1* were selected for evaluation using semi-quantitative RT-PCR. The mRNA expression of those target genes was consistent with the hybridization data for each of the genes measured. That is, mRNA levels of p21^cip1^ and ID2 were up-regulated (upper panel), while mRNA levels of RPA1, ERCC5, and GMSF were down-regulated in FN1BP1 over-expressed cells (lower panel). The statistical result of the semi-quantitative RT-PCR of the selected genes is shown in (B).

Alteration in gene expression on Hep3B Tet-On FN1BP1/S11 cells was assessed after Dox induction for 24 h. The data show that, compared with non-Dox Heb3B cells, 19 genes were up-regulated (Table 2) and 22 genes were down-regulated (Table 3) more than twofold in Dox-induced FN1BP1 expressing Hep3B cells. Of these gene changes and their putative functions, which were up-regulated compared with those of the non- Dox-induced group, most were cell-cycle–arrest proteins (p21^cip1^, p15, and cyclin E1), transcription factors (general transcription factors, zinc finger proteins, and transcriptional enhancer factors), SWItch/Sucrose NonFermentable (SWI/SNF) complex units, early-response proteins, and nerve growth or neurotrophic factors. On the other hand, down-regulated genes were subject to colony-stimulating factors (e.g., GMSF) and receptors, many repair genes after DNA damage (e.g., RAD, ERCC, DNA topoisomerase, polymerase, and ligase). Some genes (e.g., *p21^cip1^*, *ID2*, *GMSF*, *ERCC5*, and *RPA*), which that changed in the cDNA microarray analysis, were confirmed by semi-qRT-PCR, and similar changes in expression were observed (Fig. 4).

**Table 2:** Genes increased in Hep3B-tet on-FN1BP1/S11 cells induced by Dox (* selected to identify by RT-PCR).

Site	GenBank	Gene name	S11+/S11−
A7n	U17075	Cyclin-dependent kinase inhibitor 2B (p15, inhibits CDK4)	2.26
*D6f	U09579	Cyclin-dependent kinase inhibitor 1A (p21, Cip1)	2.17
A6m	M73812	CyclinE1	2.23
*D1g	M97796	Inhibitor of DNA binding 2, dominant negative helix-loop-helix protein	2.71
D4f	M62829	Early growth response 1	4.98
D4g	M62831	Immediate early protein	2.80
D1i	M88163	SWI/SNF related, matrix associated, subfamily a, member 1	4.49
D1m	L34673	SWI/SNF related, matrix associated, subfamily a, member 3	2.49
D5h	M97287	Special AT-rich sequence binding protein 1 (binds to nuclear matrix/scaffold-associating DNA’s)	2.03
D1n	M28372	Zinc finger protein 9 (a cellular retroviral nucleic acid binding protein)	2.82
D5d	M92843	Zinc finger protein 36, C3H type, homolog (mouse)	2.38
D1k	L04282	Zinc finger protein 148 (pHZ-52)	3.34
B2k	D26309	LIM domain kinase 1	2.28
D1f	M95809	General transcription factor IIH, polypeptide 1 (62 kD subunit)	3.84
D2b	Z30094	General transcription factor IIH, polypeptide 2 (44 kD subunit)	2.80
F3m	M60828	Fibroblast growth factor 7 (keratinocyte growth factor)	3.53
F2j	L12261	Neuregulin 1	2.40
F3n	M61176	Brain-derived neurotrophic factor	3.84
Fm	X13967	Leukemia inhibitory factor (cholinergic differentiation factor)	3.72

**Table 3:** Genes decreased in Hep3B-tet on-FN1BP1/S11 cells induced by Dox (* selected to identify by RT-PCR).

Site	GenBank	Gene name	S11+/S11-
A1b	X03663	Colony stimulating factor 1 receptor, formerly McDonough feline sarcoma viral (v-fms) oncogene homolog	0.34
E2i	X17648	Colony stimulating factor 2 receptor, alpha, low-affinity (granulocyte-macrophage)	0.42
*F1c	M11220	Colony stimulating factor 2 (granulocyte-macrophage)	0.32
E1m	M59818	Colony stimulating factor 3 receptor (granulocyte)	0.23
C5i	D13804	RAD51 homolog (RecA homolog, *E. coli*) (*S. cerevisiae*)	0.22
C7d	D21090	RAD23 homolog B (*S. cerevisiae*)	0.37
C7e	D21235	RAD23 homolog A (*S. cerevisiae*)	0.37
C7i	U12134	RAD52 homolog (*S. cerevisiae*)	0.08
C7k	U63139	RAD50 homolog (*S. cerevisiae*)	0.07
C6f	M74524	Ubiquitin-conjugating enzyme E2A (RAD6 homolog)	0.38
*C5m	L20046	Excision repair cross-complementing rodent repair deficiency, complementation group 5 (xeroderma pigmentosum, complementation group G (Cockayne syndrome))	0.44
C5j	L04791	Excision repair cross-complementing rodent repair deficiency, complementation group 6	0.10
C6n	X52221	Excision repair cross-complementing rodent repair deficiency, complementation group 2 (xeroderma pigmentosum D)	0.27
C7a	M13194	Excision repair cross-complementing rodent repair deficiency, complementation group 1 (includes overlapping antisense sequence)	0.27
C5k	L07540	Replication factor C (activator 1) 5 (36.5 kD)	0.14
*C6e	M63488	Replication protein A1 (70 kD)	0.46
C6g	M87338	Replication factor C (activator 1) 2 (40 kD)	0.20
C6d	M36089	X-ray repair complementing defective repair in Chinese hamster cells 1	0.11
C6i	X06745	Polymerase (DNA directed), alpha	0.28
C6k	J03250	Topoisomerase (DNA) I	0.10
C7l	X83441	Ligase IV, DNA, ATP-dependent	0.20
C7m	X84740	Ligase III, DNA, ATP-dependent	0.10

### 5 FN1BP1 Caused More Hep3B Cells Arrested in G1 Phase

**Figure 5:**
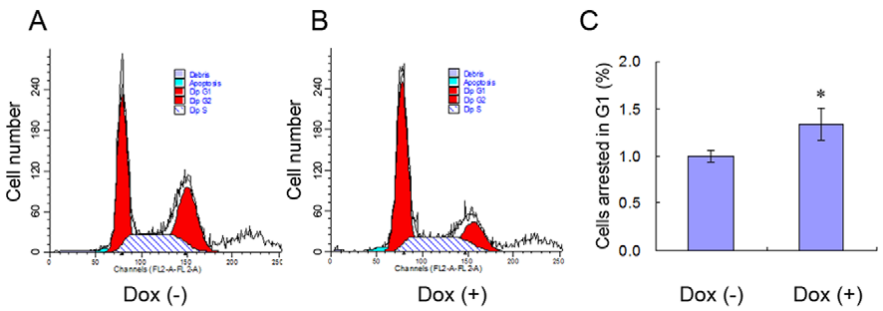
The expression of FN1BP1 induced G1 phase arrest. Synchronized by nocodazole for 20 h, Dox was added to half of the plates to induce the expression of FN1BP1 for 24 h. Following UV irradiation at 200 (×100 µJ/cm2) for 1 min, the cells were obtained after another 12-h incubation. Then the cells were collected and stained by propidium iodide (PI) for flow cytometry (FCM). (A–B) showed the representative figure of the result of FCM for DOX (−) and DOX (+), respectively. The statistical result is shown in (C). The statistical data demonstrate that more cells induced by Dox were arrested in the G1 phase compared with the non-Dox–induced cells. *P<0.05, n = 5.

As indicated by Atlas cDNA microarray, some cell-cycle–arrest genes (*p21^cip1^*, *p15*, and cyclin E1) were up-regulated, while many repair genes (e.g., RAD, ERCC, DNA topoisomerase, polymerase, and ligase) were down-regulated when FN1BP1 expression was induced. Because these genes are involved in DNA repair after damage [17], [18] and cell cycle arrest [2], [19], FCM was performed to investigate the effect of FN1BP1 expression on cell cycle in Hep3B cells after the synchronization of nocodazole and UV irradiation. The result of FCM analysis showed that, compared with the non-induced cells, the Dox-induced FN1BP1 over-expression arrested 134±17% of Hep3B cells in the G1 phase (Fig. 5).

## Discussion

The experiments performed in this study demonstrate the characteristics and function of the *FN1BP1* gene in hepatocarcinoma Hep3B cells. As a novel human gene, we chose multiple human tissues to investigate the FN1BP1 mRNA expression pattern, and we found that a ∼2.8-kb fragment existed in human placenta, liver and skeletal muscle tissues. The length of this mRNA transcript was similar to the full FN1BP1 sequence we had obtained previously (GenBank Accession No. AF217970.1). Because the ORF sequence of FN1BP1 was postulated to be a secreted protein with a signal peptide by the online SignalP software, we collected the supernatant of cultured cell media from Hep3B cells to trace the FN1BP1 expression using western blotting assay. We found that, after over-expression for 72 h, FN1BP1 protein accumulation in the culture media could be detected. Meanwhile, the FN1BP1 protein-fused green fluorescent protein (GFP) was observed in the cytoplasm; it exhibited granules, which might indicate that the protein was packed in some vesicles.

The tight temporal control of gene expression is a very useful tool in basic biological applications. Several conditional expression systems have been developed, and currently the tetracycline (Tet)-regulated gene expression system is widely utilized [20], [21], [22]. Therefore, we established a conditionally induced stable cell line of Hep3B-Tet-on-FN1BP1/S11 using the Tet-On induction system to investigate the preliminary function and mechanism of the FN1BP1 protein, in which the *FN1BP1* gene could be conditionally induced by Dox. Our results demonstrate that FN1BP1 expression reduces cell proliferation and colony formation of Hep3B cells. The results of our Atlas human cDNA expression array for general gene function indicate that after FN1BP1 Dox-induced expression for 24 h, 19 genes were up-regulated and 22 genes were down-regulated more than two-fold. Of these gene changes and their putative functions, which were up-regulated compared with the non-induced group, most were cell-cycle–arrest proteins (e.g., p21cip1, p15, and cyclin E1), transcription factors (e.g., general transcription factors, zinc finger proteins, and transcriptional enhancer factors), SWI/SNF complex units, early-response proteins, and nerve growth or neurotrophic factors. On the other hand, down-regulated genes were subject to colony-stimulating factors (e.g., GMSF), many repair genes involved in DNA damage (e.g., RAD, ERCC, DNA topoisomerase, polymerase, and ligase). Some genes (*p21cip1*, *ID2*, *GMSF*, *ERCC5,* and *RPA1*), which changed in the cDNA microarray analysis, were confirmed by semi-qRT-PCR, and similar changes in expression were observed. According to the Atlas microarray assay, most of these genes were involved in DNA repair after damage [17], [18] and cell-cycle arrest [2], [19]. We performed the cell-cycle analysis using FCM; our results indicated that FN1BP1 over-expression could result in G1 phase arrest. In addition, SWI/SNF complex units were up-regulated. Growing genetic and molecular evidence from recent studies indicates that subunits of the SWI/SNF complex act as tumor suppressors in humans and mice [23], [24], [25], [26]. These experiments may provide insight into the molecular mechanisms underlying SWI/SNF function in tumor suppression as well as the function of FN1BP1. The relationship between the *FN1BP1* gene and SWI/SNF warrants further research.

In summary, these studies of *FN1BP1*, a novel gene identified using the human placenta cDNA library in our laboratory, show that this gene can inhibit cell growth and colony conformation through G1 phase arrest of the Hep3B cell cycle. The results of this study indicate the potential role of FN1BP1 as a treatment target for hepatocellular carcinoma.
